# Supplementary figures and images for: Treating intrusive memories after trauma in healthcare workers: a Bayesian adaptive randomised trial developing an imagery-competing task intervention
Source: Mol Psychiatry. 2023 Apr 26;28(7):2985–94. doi: 10.1038/s41380-023-02062-7 (PMC10131522; doi:10.1038/s41380-023-02062-7)

Arm Delayed Immediate

A: Baseline

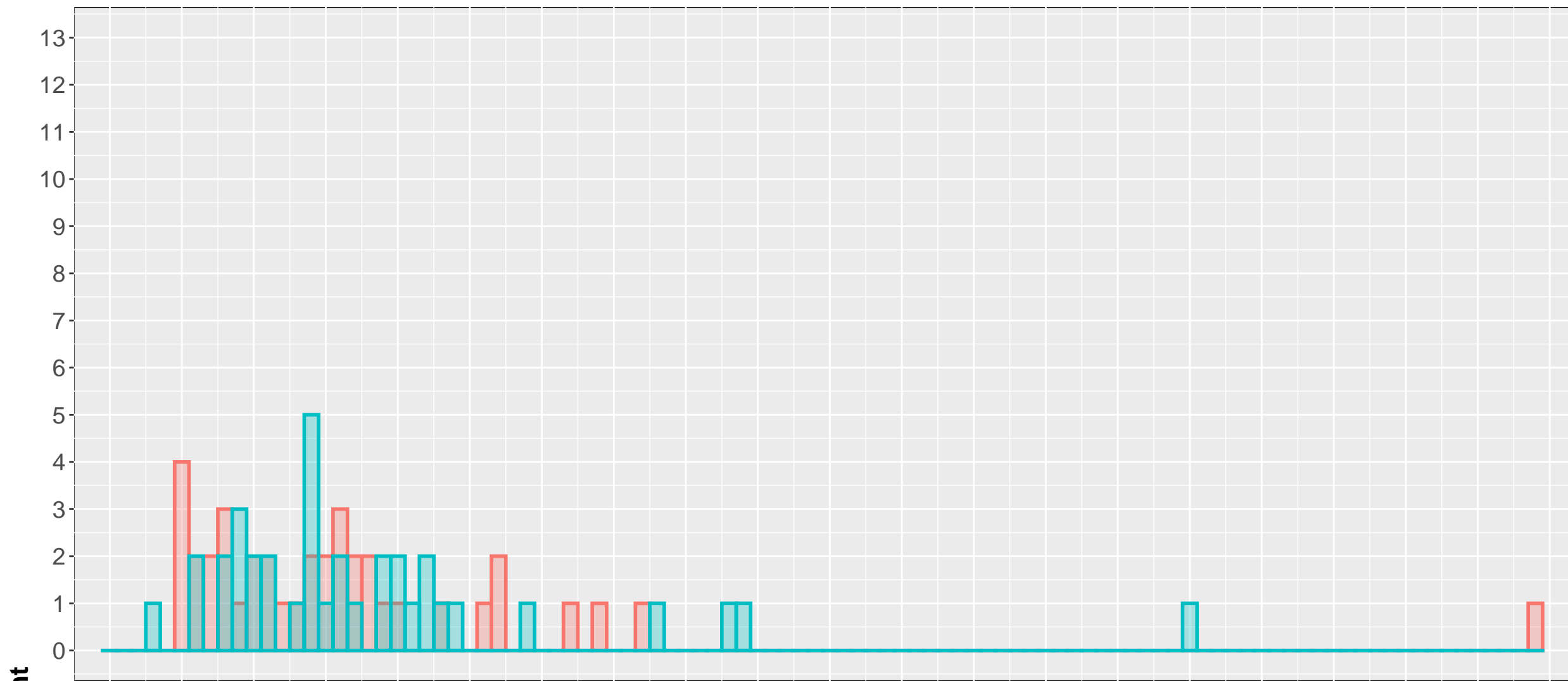

B: Week 4

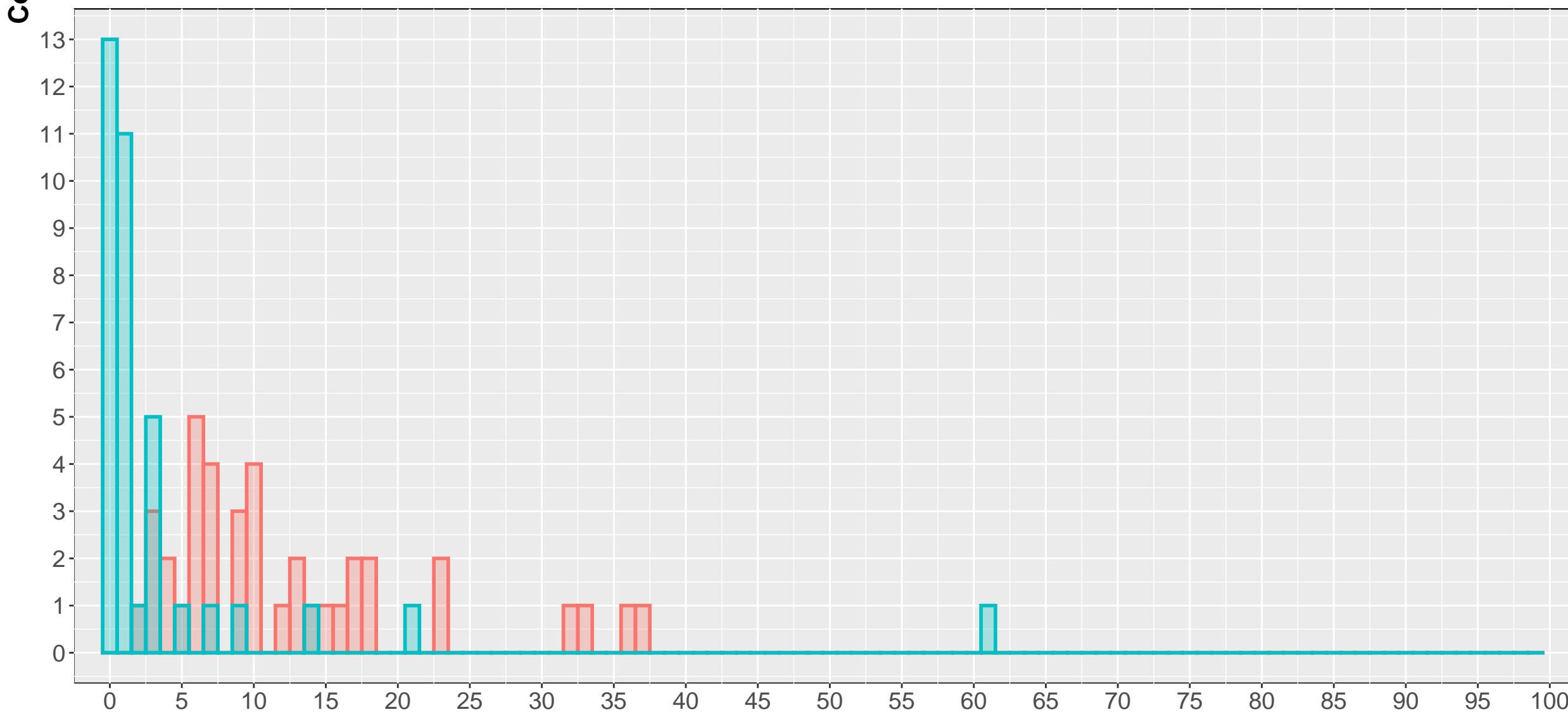

Number of Intrusive Memories in diary for 7 days

Supplement: Supplementary file 2 — Supplementary Figure 1: Primary Outcome Histograms. [file 41380_2023_2062_MOESM2_ESM.pdf]

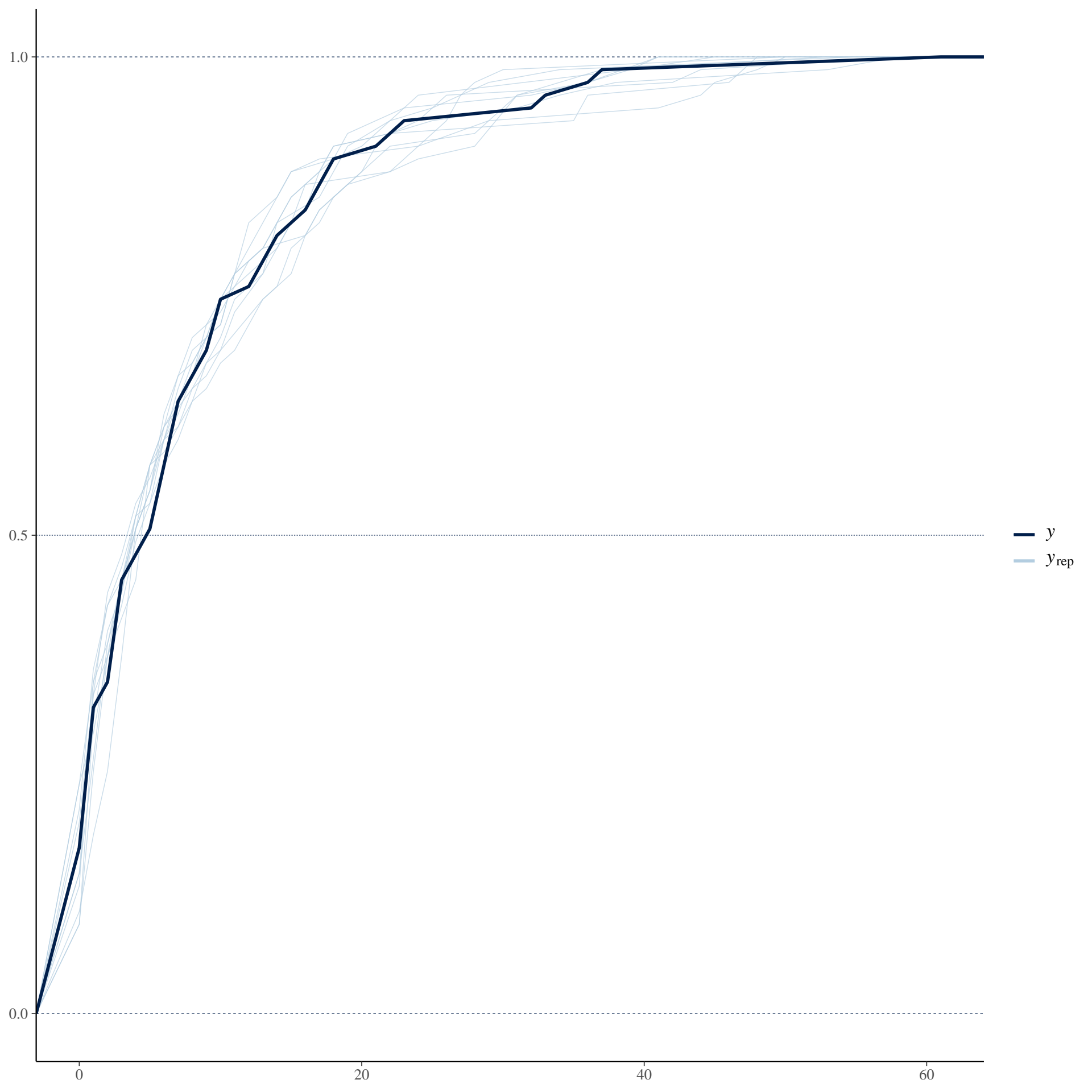

Supplement: Supplementary file 4 — Supplementary Figure 3: Posterior Predictive Checks. [file 41380_2023_2062_MOESM4_ESM.pdf]

**A**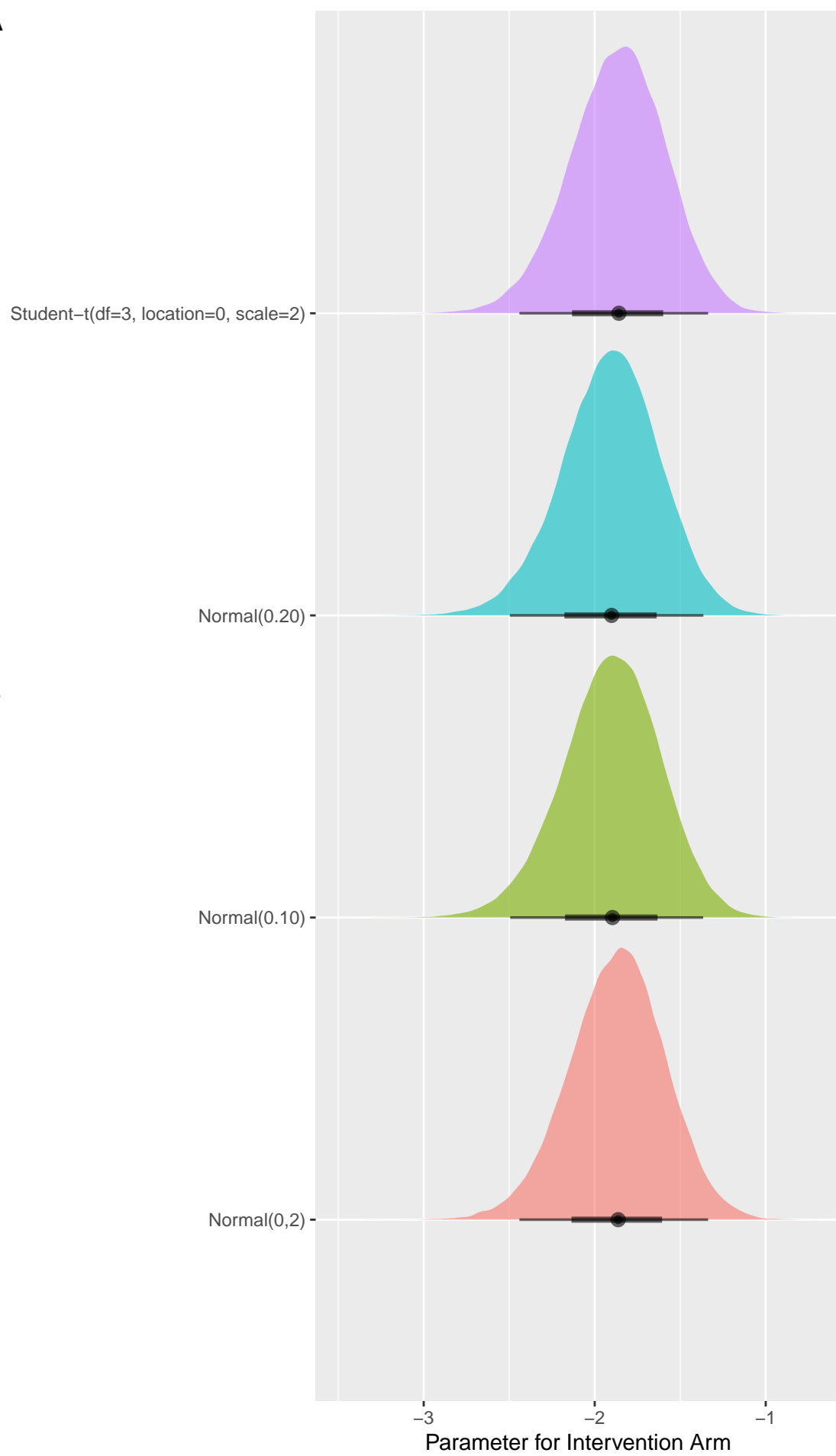**B**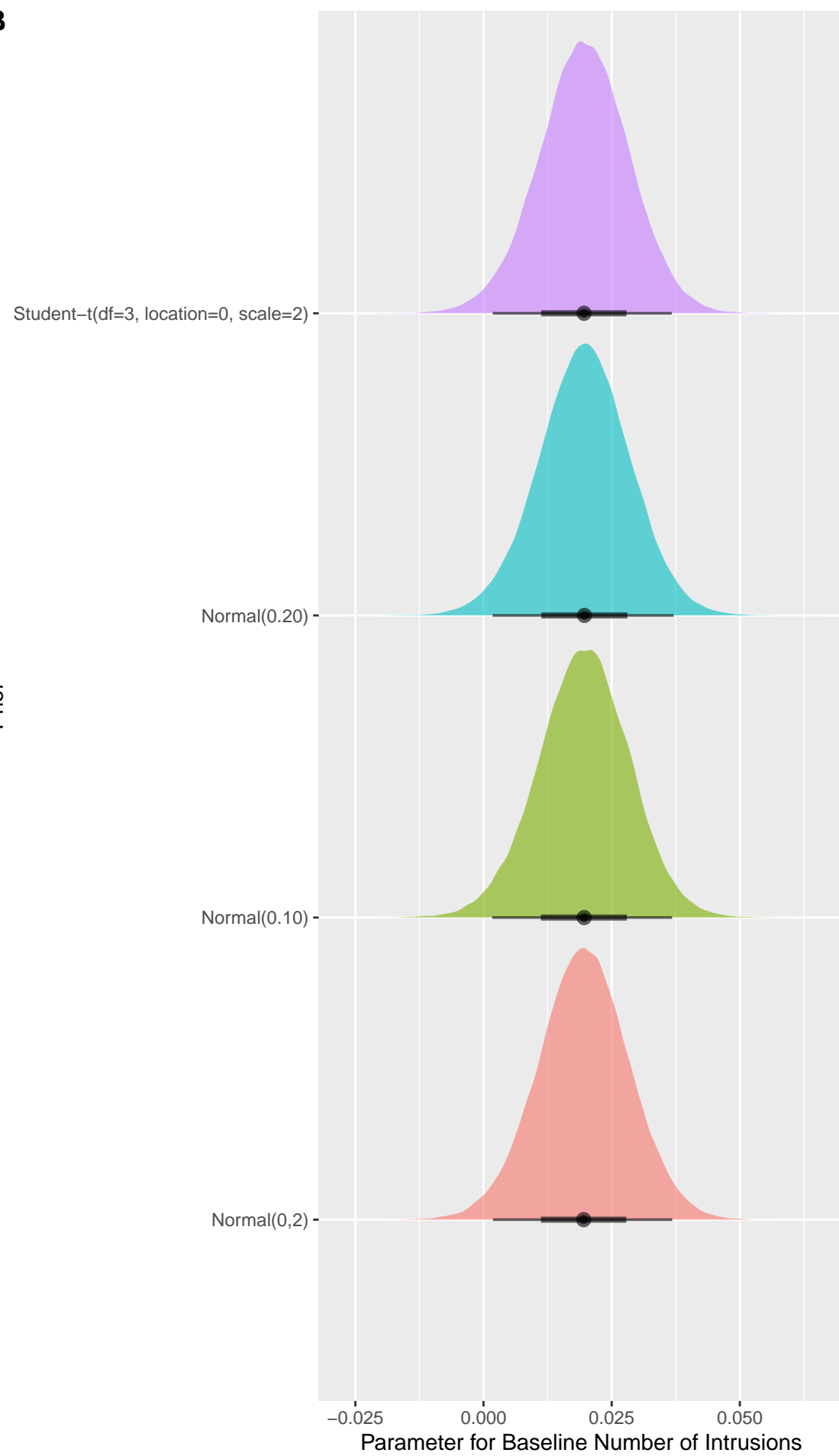

Supplement: Supplementary file 5 — Supplementary Figure 4: Posterior Density Plots for Varying Priors. [file 41380_2023_2062_MOESM5_ESM.pdf]

**A**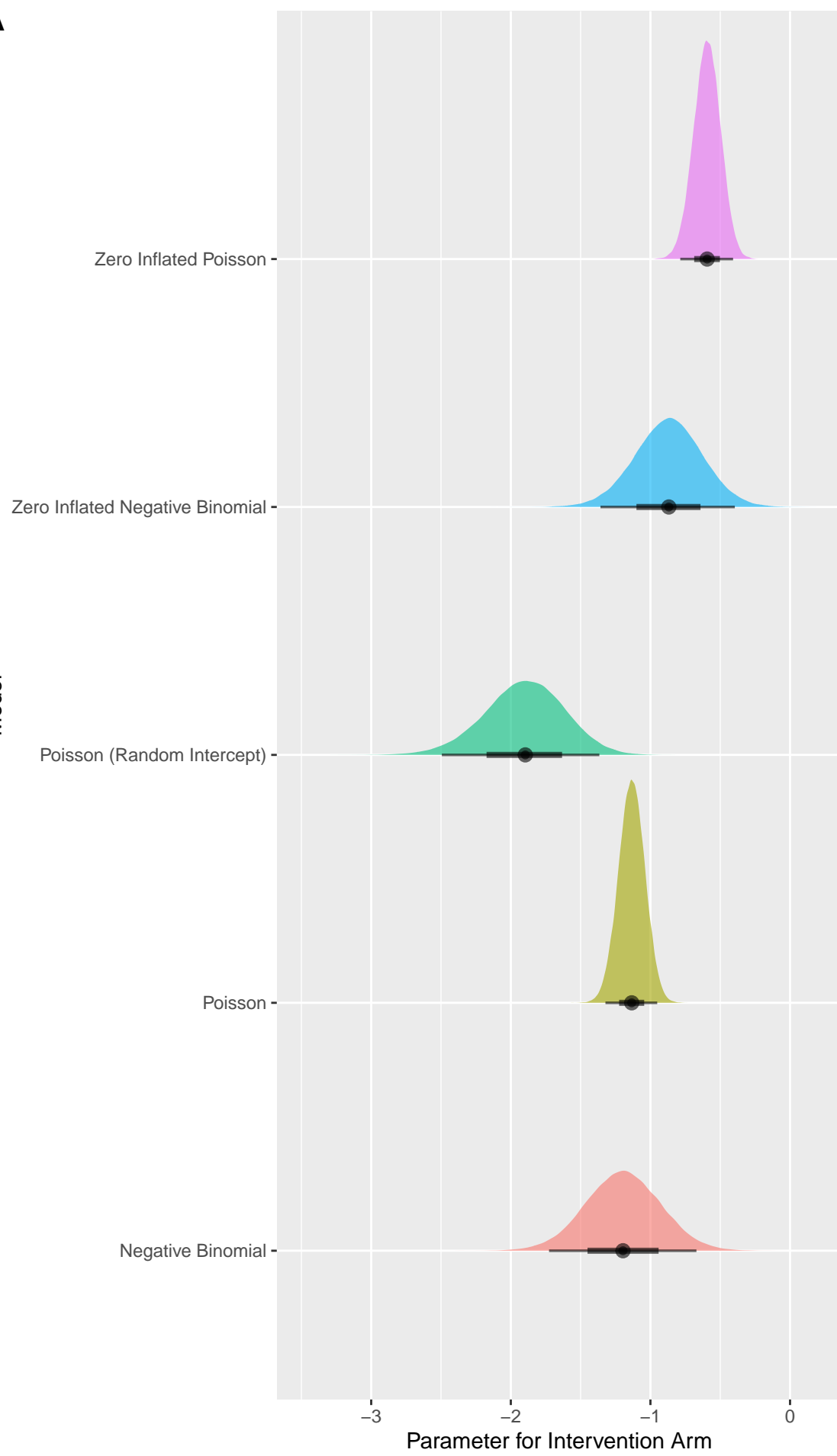**B**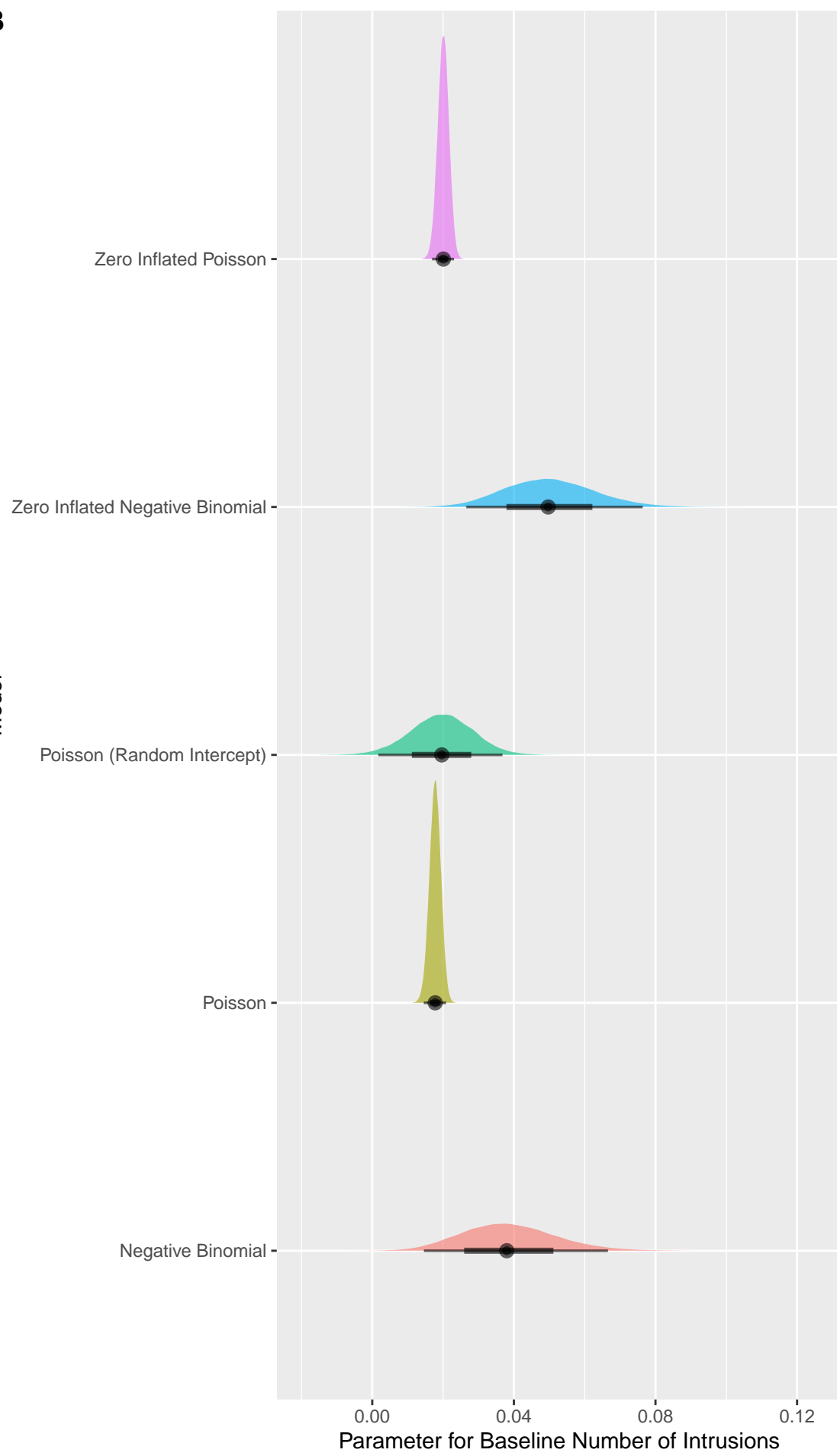

Supplement: Supplementary file 6 — Supplementary Figure 5: Posterior Density Plots for Varying Models. [file 41380_2023_2062_MOESM6_ESM.pdf]

**A**

Data Without Outliers

All Data

Data

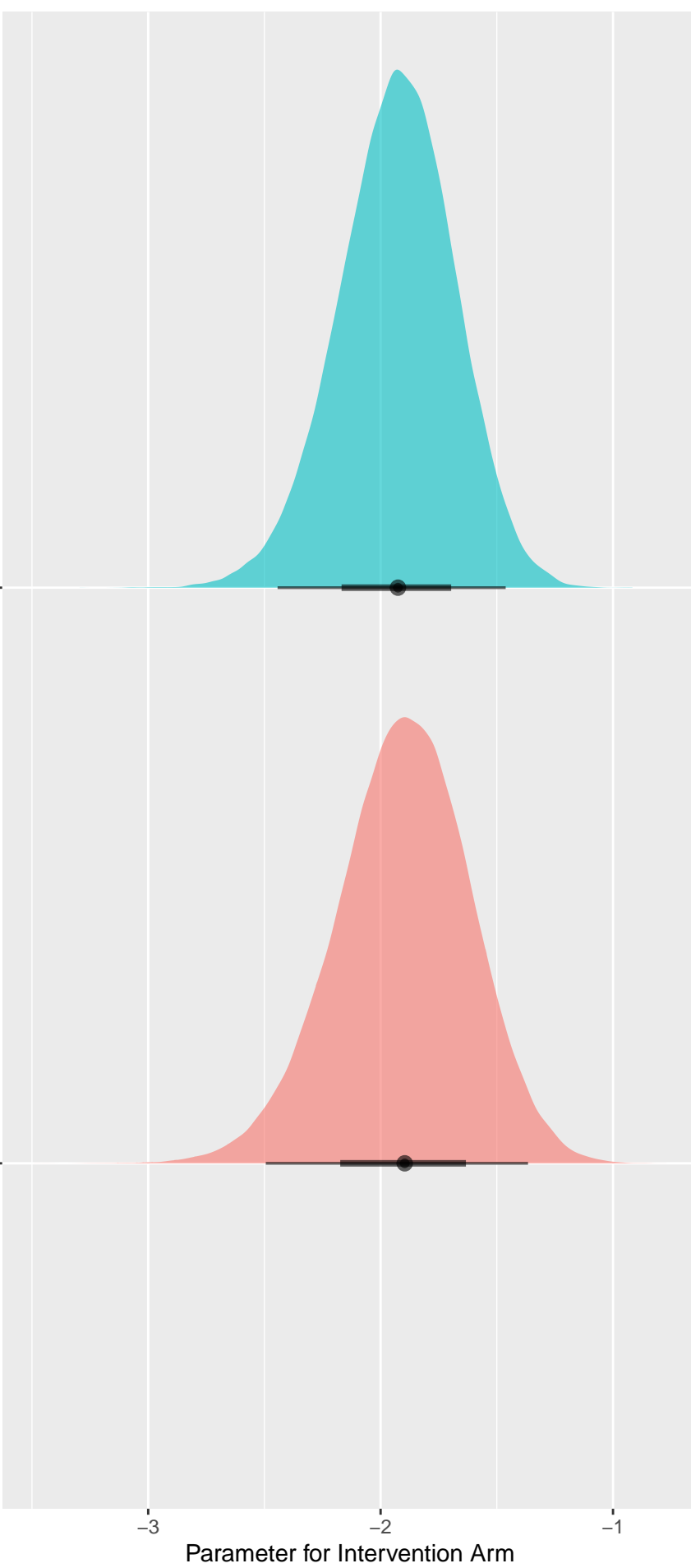**B**

Data Without Outliers

All Data

Data

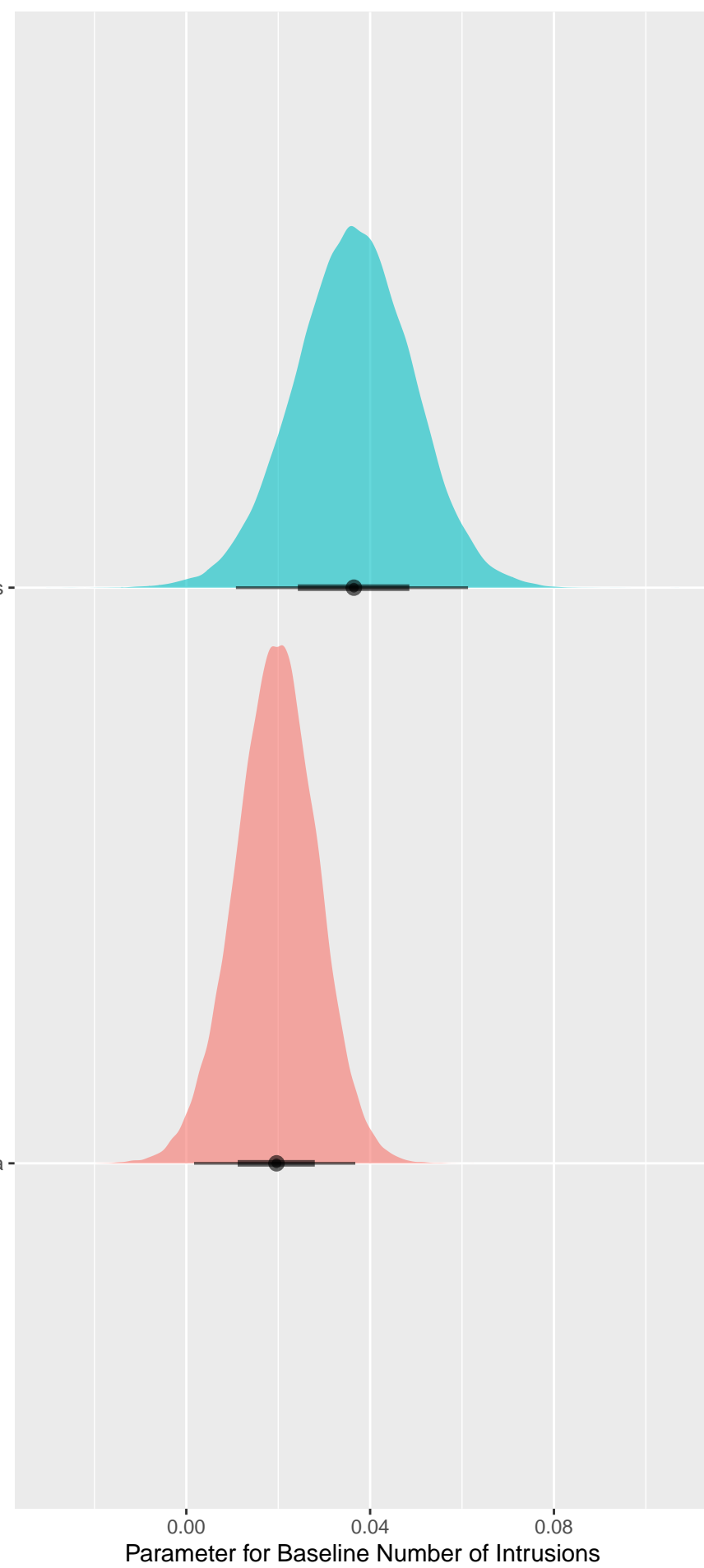

Supplement: Supplementary file 7 — Supplementary Figure 6: Posterior Density Plots for Data With and Without Outliers. [file 41380_2023_2062_MOESM7_ESM.pdf]

Bayes Factor vs Sample Size: Testing for Superiority  
of immediate arm vs delayed arm

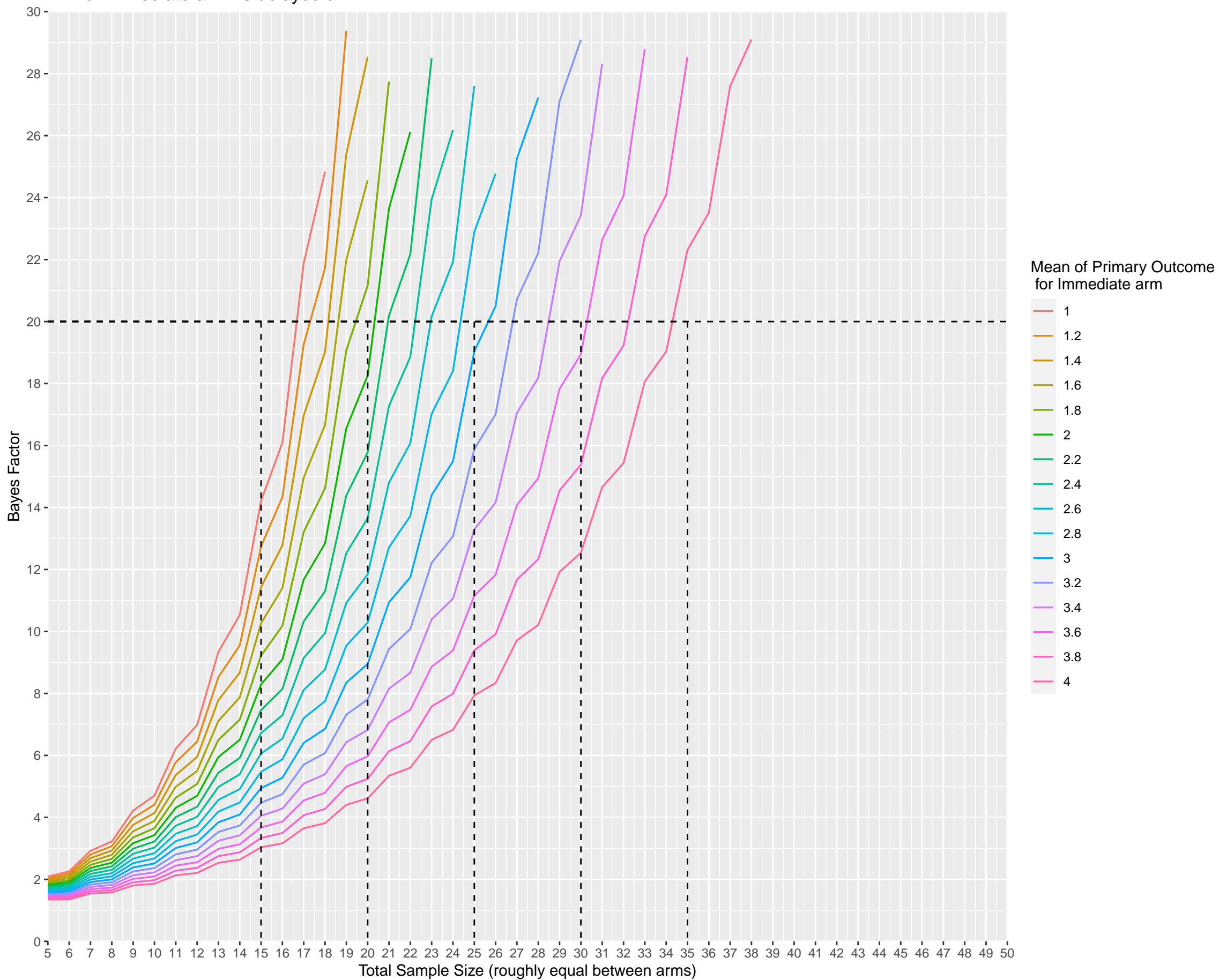

Supplement: Supplementary file 9 — Supplementary Figure 8: Bayes Factor vs Sample Size plot to Test for a Positive Treatment Effect under the Optimised Intervention. [file 41380_2023_2062_MOESM9_ESM.pdf]
