# Supplementary material for: Treating intrusive memories after trauma in healthcare workers: a Bayesian adaptive randomised trial developing an imagery-competing task intervention
Source: Mol Psychiatry. 2023 Apr 26;28(7):2985–94. doi: 10.1038/s41380-023-02062-7 (PMC10131522; doi:10.1038/s41380-023-02062-7)
Supplement: Supplementary file 3 — Supplementary Figure 2: Posterior Trace and Density Plots. [file 41380_2023_2062_MOESM3_ESM.pdf]

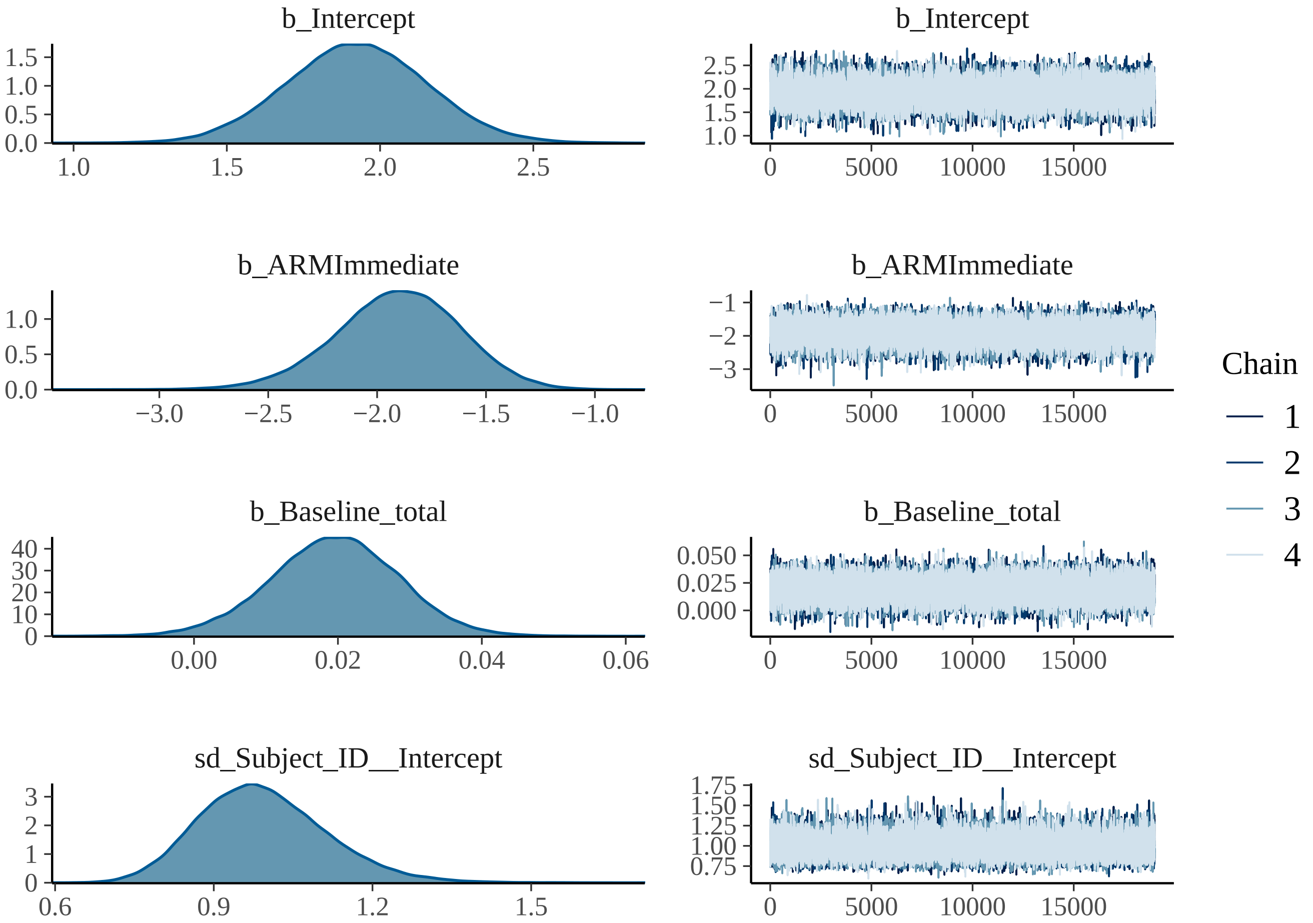
b_Intercept
1.0
1.5
2.0
2.5
b_ARMImmediate
−3.0
−2.5
−2.0
−1.5
−1.0
b_Baseline_total
0.00
0.02
0.04
0.06
sd_Subject_ID__Intercept
0.6
0.9
1.2
1.5
0
5000
10000
15000
Chain
1
2
3
4
