# Supplementary material for: Treating intrusive memories after trauma in healthcare workers: a Bayesian adaptive randomised trial developing an imagery-competing task intervention
Source: Mol Psychiatry. 2023 Apr 26;28(7):2985–94. doi: 10.1038/s41380-023-02062-7 (PMC10131522; doi:10.1038/s41380-023-02062-7)
Supplement: Supplementary file 8 — Supplementary Figure 7: Posterior Density Plots for All Data and Non-imputed Data. [file 41380_2023_2062_MOESM8_ESM.pdf]

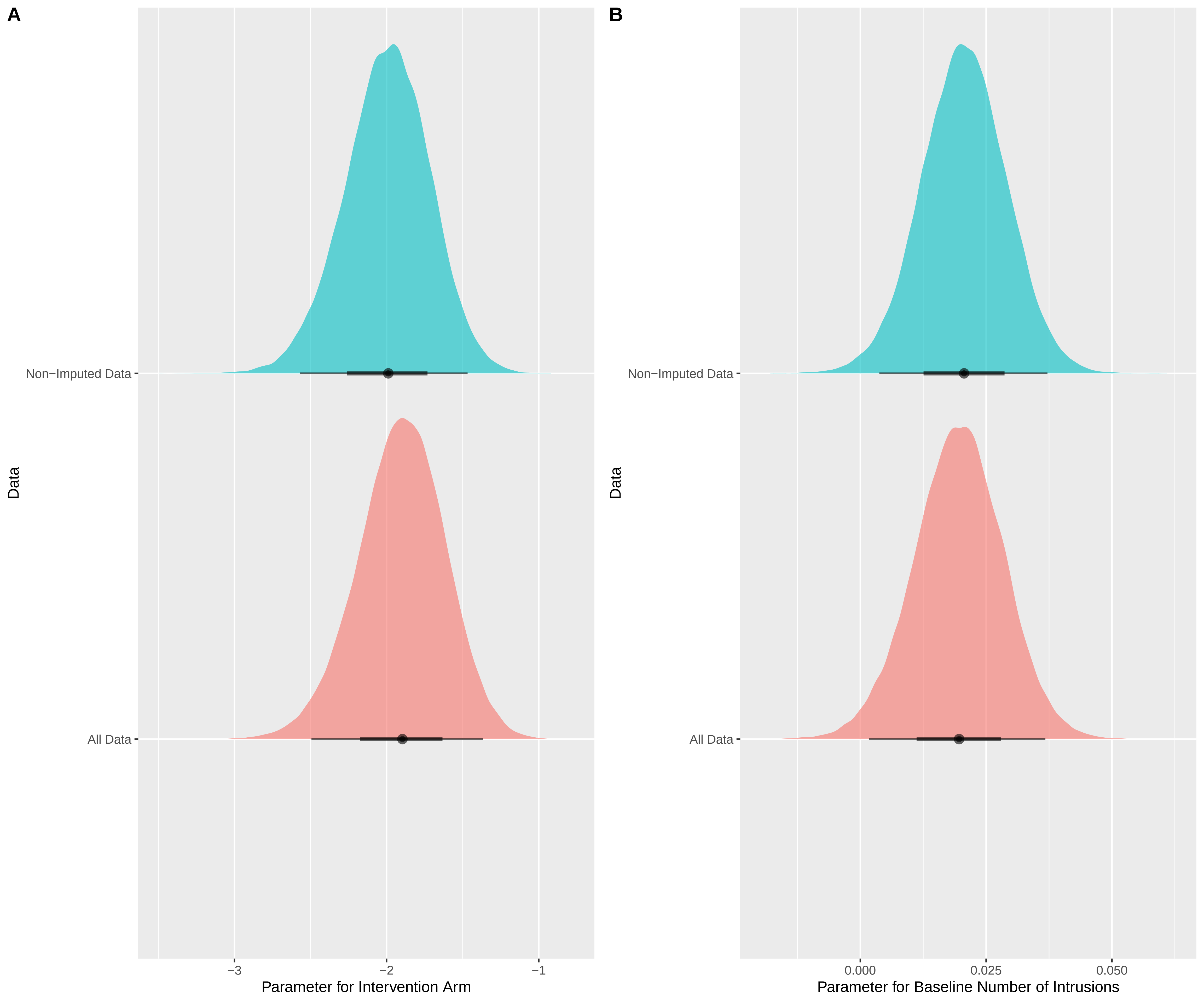
A
B
Non−Imputed Data
All Data
Data
−3
−2
−1
Parameter for Intervention Arm
0.000
0.025
0.050
Parameter for Baseline Number of Intrusions
