# Supplementary material for: Treating intrusive memories after trauma in healthcare workers: a Bayesian adaptive randomised trial developing an imagery-competing task intervention
Source: Mol Psychiatry. 2023 Apr 26;28(7):2985–94. doi: 10.1038/s41380-023-02062-7 (PMC10131522; doi:10.1038/s41380-023-02062-7)
Supplement: Supplementary file 10 — Supplementary Figure 9: Bayes Factor vs Sample Size plot to Compare the Optimised Intervention to the Former Un-optimised Intervention. [file 41380_2023_2062_MOESM10_ESM.pdf]

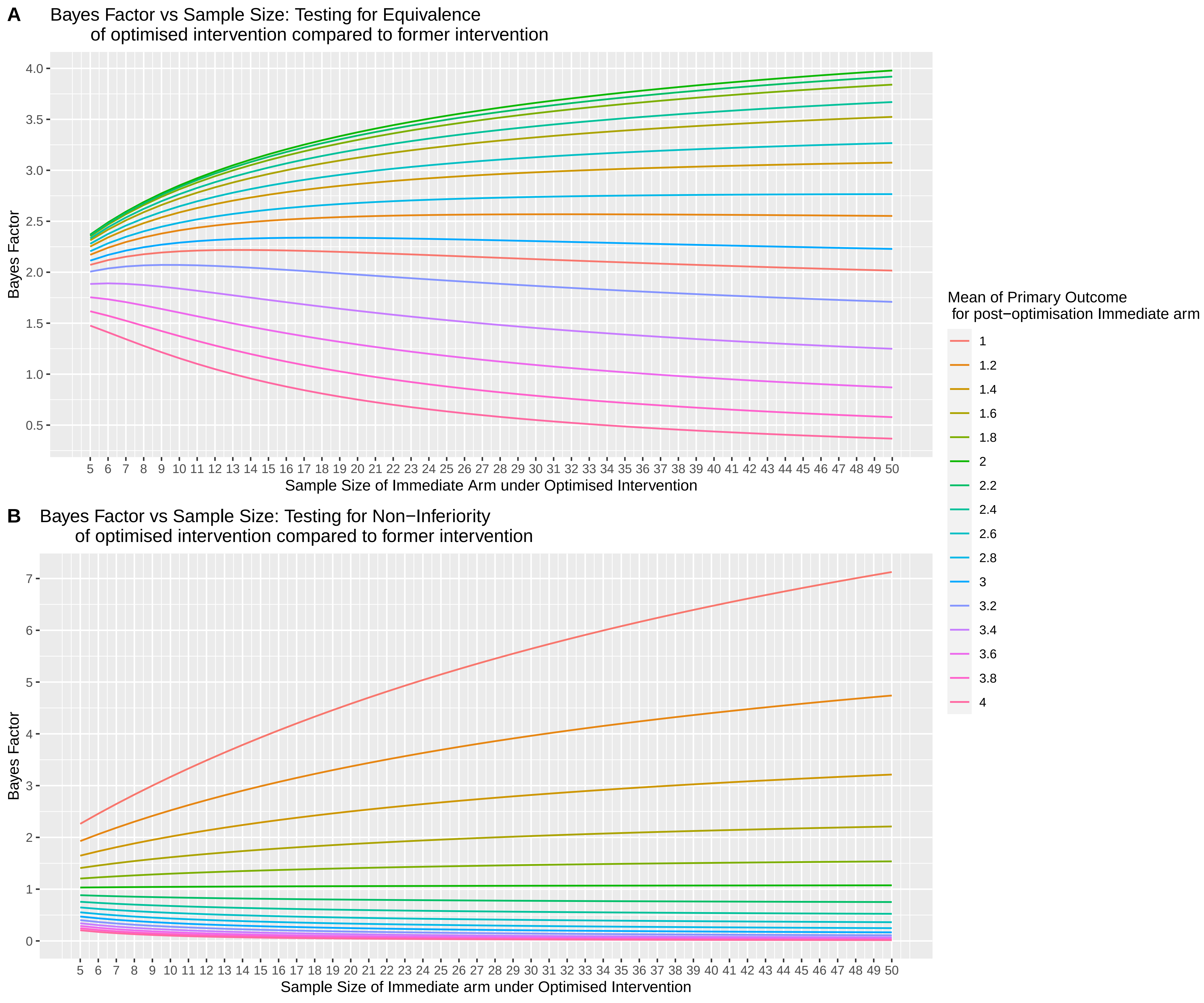
A
Bayes Factor vs Sample Size: Testing for Equivalence
of optimised intervention compared to former intervention
Bayes Factor
4.0
3.5
3.0
2.5
2.0
1.5
1.0
0.5
5 6 7 8 9 10 11 12 13 14 15 16 17 18 19 20 21 22 23 24 25 26 27 28 29 30 31 32 33 34 35 36 37 38 39 40 41 42 43 44 45 46 47 48 49 50
Sample Size of Immediate Arm under Optimised Intervention
B
Bayes Factor vs Sample Size: Testing for Non−Inferiority
of optimised intervention compared to former intervention
Bayes Factor
7
6
5
4
3
2
1
0
5 6 7 8 9 10 11 12 13 14 15 16 17 18 19 20 21 22 23 24 25 26 27 28 29 30 31 32 33 34 35 36 37 38 39 40 41 42 43 44 45 46 47 48 49 50
Sample Size of Immediate arm under Optimised Intervention
Mean of Primary Outcome
for post−optimisation Immediate arm
1
1.2
1.4
1.6
1.8
2
2.2
2.4
2.6
2.8
3
3.2
3.4
3.6
3.8
4
